# Citizen advisory groups for the creation and improvement of decision aids: experience from two Swiss centers for primary care

**DOI:** 10.1186/s40900-021-00283-0

**Published:** 2021-06-05

**Authors:** Kevin Selby, Regula Cardinaux, Beatrice Metry, Simone de Rougemont, Janine Chabloz, Verena Meier-Herrmann, Jürg Stoller, Marie-Anne Durand, Reto Auer

**Affiliations:** 1 Center for primary care and public health (Unisanté), University of Lausanne, Rue de Bugnon 44, 1010 Lausanne, Switzerland; 2 Institute of primary health care (BIHAM), University of Bern, Bern, Switzerland; 3 Lausanne, Switzerland; 4 Ecublens, Switzerland; 5 Mörigen, Switzerland; 6 Münsingen, Switzerland; 7 University of Toulouse, Toulouse, France; 8 The Dartmouth Institute for Health Policy and Clinical Practice, Dartmouth College, Lebanon, NH USA

**Keywords:** Decision aids, Participatory research, Citizen involvement

## Abstract

**Background:**

Guidelines for patient decision aids (DA) recommend target population involvement throughout the development process, but developers may struggle because of limited resources. We sought to develop a feasible means of getting repeated feedback from users.

**Methods:**

Between 2017 and 2020, two Swiss centers for primary care (Lausanne and Bern) created citizen advisory groups to contribute to multiple improvement cycles for colorectal, prostate and lung cancer screening DAs. Following Community Based Participatory Research principles, we collaborated with local organizations to recruit citizens aged 50 to 75 without previous cancer diagnoses. We remunerated incidental costs and participant time. One center supplemented in-person meetings by mailed paper questionnaires, while the other supplemented meetings using small-group workshops and analyses of meeting transcripts.

**Results:**

In Lausanne, we received input from 49 participants for three DAs between 2017 and 2020. For each topic, participants gave feedback on the initial draft and 2 subsequent versions during in-person meetings with ~ 8 participants and one round of mailed questionnaires. In Bern, 10 participants were recruited among standardized patients from the university, all of whom attended in-person meetings every three months between 2017 and 2020. At both sites, numerous changes were made to the content, appearance, language, and tone of DAs and outreach materials. Participants reported high levels of satisfaction with the participative process.

**Conclusions:**

Citizen advisory groups are a feasible means of repeatedly incorporating end-user feedback during the creation of multiple DAs. Methodological differences between the two centers underline the need for a flexible model adapted to local needs.

**Supplementary Information:**

The online version contains supplementary material available at 10.1186/s40900-021-00283-0.

## Plain English summary

Decision aids are paper or electronic tools that help people make medical decisions. They have been shown to help with shared decision making between patients, their loved ones, and a health professional. When we create decision aids, we try to involve patients at each step, traditionally using focus groups. However, new approaches are needed because focus groups take a lot of time and money.

We developed a new method of involving people eligible for cancer screening and used this method during the development of four decision aids at two centers for research in primary care. Two of the decision aids were for colorectal cancer screening (one in French, one in German), and one each for prostate and lung cancer screening. We recruited people aged 50 to 75 interested in improving health information materials from community organizations and among standardized patients from local medical schools to form citizen advisory groups. Standardized patients act as patients during teaching sessions and exams. Some people took part in face-to-face meetings to give us feedback on the materials, while others responded to questionnaires and gave feedback by mail. The same participants gave feedback multiple times as we made improvements to the decision aids.

The citizen advisory groups provided us with repeated, meaningful input during the development of decision aids. We think they allowed us to create better, more patient-centered decision aids, while using fewer resources than traditional focus groups. Other researchers who develop decision aids may want to use a similar approach.

**Supplementary Information:**

The online version contains supplementary material available at 10.1186/s40900-021-00283-0.

## Introduction

Large practice variations between physicians suggest that their preferences often dominate those of patients when making preference-sensitive decisions [1]. Shared decision making (SDM) can help clinicians better incorporate patient preferences [2, 3], a core component of patient-centered care [4]. Decision aids (DAs) support SDM by making decisions explicit, providing reliable information about the benefits and harms of available options, and clarifying which choices fit with individual preferences [5]. Over 100 randomized trials have shown that DAs increase knowledge, decrease anxiety related to choices, and improve decision making [5].

International guidelines for the creation of DAs specify that their development process should include a needs assessment, review, and testing by clients or patients targeted by the materials [6, 7]. Several established DA developers do extensive user-testing to ensure their DAs fit into routine patient and provider interactions [8]. However, these approaches are very resource intensive for smaller developers, especially if new users need to be recruited to test each iteration of a DA or DAs addressing multiple health issues in a short period of time. In our experience, most DA developers primarily involve end users in focus groups either for an initial needs assessment or at the end of development for validation of content and messages. While focus groups are a cost-effective means of collecting qualitative data, small research groups do not have sufficient resources to allow for testing of alternate versions of the DA or smaller changes. New models are needed if we want to have DAs be widely available for a large number of treatment decisions [9].

An alternative to focus groups would be to recruit a pool of people from the target population who can be solicited on multiple occasions, borrowing from the principles of Community-Based Participatory Research [10]. The same members could meet regularly, similar to a Community Advisory Board, though their role would be to provide regular feedback on materials rather than serve a leadership role in the research project. Repeated meetings could allow iterative feedback, similar to plan-do-study-act cycles used in quality improvement [11]. This approach would save time spent on recruitment and allow for shorter meetings as citizens acquire expertise over time.

This paper describes the experience of two Swiss academic primary care centers who formed and maintained citizen advisory groups. Our goal was to get repeated feedback during the creation and improvement of materials for cancer screening. We hypothesized that such groups could provide relevant, ongoing feedback in the development of patient materials.

## Methods

### Setting

This study took place in two Swiss university centers for primary care that develop materials for SDM. The Center for Primary Care and Public Health (Unisanté) in Lausanne works with local partners to provide DAs about cancer screening. The Bern citizen advisory group was formed in the context of a cluster randomized trial funded by the Swiss National Science Foundation about patient choice in CRC screening. The trial promoted shared decision making between general practitioners and patients for invasive and non-invasive options for CRC screening [12]. Principal differences in the methods used by each center are summarized in Table 1.

**Table 1:** Differences in approach between Bern and Lausanne

Element	Lausanne Model	Bern Model
Initial inclusion in the group	Individual recruitment via community partners. No contract or informed consent	Initial information session, followed by contract and informed consent
Group composition	59% female, mean age 62 years. Patients from our local academic practice (30%), local consumer organization (18%), an association for the elderly (10%), standardized patients (10%), and personal contacts of other participants or the researchers (32%)	70% female, mean age 65 years. Standardized patients (100%).
Topics discussed	Decision aids for colorectal, prostate and lung cancer screening	Multiple documents and components of colorectal cancer screening
Meeting frequency	Average three in-person meetings and two mailings per year, scheduled when materials became available	Two-hour, in-person meeting every quarter
Reimbursement	Cost of parking and public transport; 50 CHF gift certificates distributed to in-person and mail participants	30 CHF/hour salary, paid by the Swiss National Science Foundation as a one-time annual bank transfer
Information sources	In-person meetings with 6 to 10 people supplemented by mailings to an additional 30 to 40 people	In-person meetings with 7 to 10 people employing both round-table discussions and small-group workshops

*CHF* Swiss Francs

### Recruitment

In 2015, the Lausanne group conducted a one-year pilot with a small citizen advisory group recruited among standardized patients from the Lausanne medical school. Standardized patients are trained and paid by medical schools to act as patients during medical student examinations. They represent a diverse pool of people trained in multiple patient roles, providing feedback on medical communication, and empathizing with problems citizens encounter when making medical decisions [13]. In 2016 we partnered with a local organization for senior citizens and a consumer organization to recruit additional members to our group. Given our focus on cancer screening, eligibility criteria were age 50 to 75 years, no previous cancer diagnosis from the cancers being discussed, and the ability to have spoken communication in French. We did not have a specific information session, but the goals of the project were explained at the first feedback meeting. We did not get IRB approval or obtain written consent because our project was considered quality improvement.

The Bern group was started in late 2017, when an email was sent to all standardized patients at the University of Bern inviting those aged 50 to 75 with no previous diagnosis of CRC to participate in a research group. After an evening information session, 10 volunteered (seven women, three men). All participants signed contracts, after which we paid them 30 CHF (Swiss Francs) per hour of work. The salary was paid once per year directly by the Swiss National Science Foundation. This method was approved by the local institutional review board. Participants also signed an informed consent.

### Procedure

**Fig. 1:**
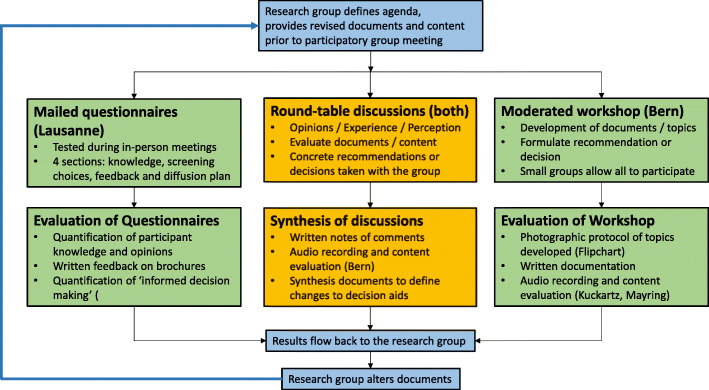
Model describing methodology used for improvement cycles in Lausanne and Bern. Squares in Blue show work by the research group outside of data collection, in yellow show elements used by both centers, while green squares show elements used in just Lausanne or just Bern

A common element was that both Lausanne and Bern used iterative improvement cycles and in-person meetings to discuss content (Fig. 1). In Lausanne, we had six in-person meetings over 2 years to assist in the development of three decisions aids: three meetings to discuss materials for colorectal cancer screening, two meetings for prostate cancer, and one for lung cancer screening (cut short by COVID-19 pandemic). Materials for review were sent to participants by post at least ten days prior. Sessions were held in the evenings with a light meal served. We reimbursed costs of public transport or parking and provided 50 CHF gift certificates after each session. Gift certificates were used to avoid administrative hurdles of having participants be employees. Two researchers attended each meeting with one acting as moderator and the other as note-taker, based on a semi-structured interview guide. Participants provided overall views on DAs, followed by a page by page review of principal messages and wording.

In addition the Lausanne group did three rounds of postal data collection using questionnaires (Fig. 1). Each French-language questionnaire had three or four sections (supplementary materials): knowledge after reading the questionnaire, screening preferences and intentions, impressions of the materials, and feedback on the participative process and means of disseminating the materials. Most answers were multiple choice. Our original goal with the written questionnaires was to measure informed decision making after reading the brochures using criteria developed for a large randomized trial [14]. We found that our small sample size and lack of comparator group made it difficult to use these data. When discussing prostate cancer screening, the in-person meeting was limited to only men to increase comfort discussing intimate topics, but both men and women were submitted mailed questionnaires. Women were asked to imagine they needed to counsel a loved one when reviewing documents. A similar approach was used for never-smokers and lung cancer.

In Bern meetings were held quarterly, with the group working mainly during meetings. There was also preparatory work between meetings that the individual did at home that was also paid at 30 CHF per hour. As a comparison, standardized patients receive 30 CHF/hour during training and preparatory work and 50 CHF/hour during student exams. We obtained feedback on CRC brochures, optimal means of administering CRC screening general practice, as well as information letters.

In Bern, both discussion groups and work in small groups (3–4 people) were used to promote the interaction between the group members and to generate as diverse an input as possible in the groups (Fig. 1). The group meetings in Bern were integrated into a randomized trial, and enabled the research group to present not just information materials, but also central aspects of their research activities. The researchers got direct feedback and insights from a group of persons representing the perspective of persons invited to CRC screening. The participants received materials to read and edit before the meetings, so that they could arrive prepared. Two researchers were at each meeting with one serving as moderator. All discussions were audio recorded, transcribed and reviewed to ensure the accuracy of conclusions. The transcripts were subjected to a basic thematic analysis. In group work in particular, aspects emerged that were not discussed in the plenum and were nevertheless considered important.

### Patient involvement in manuscript preparation

Two members of each group were asked to participate in the preparation of the manuscript. Given language barriers (Lausanne is primarily French and Bern Swiss German speaking), the abstract and portions of the main text were translated into French and German at various stages of preparation. They were also presented the Tables and Figures. The group members then gave verbal feedback that was integrated by the other authors. All authors approved the final English manuscript.

## Results

### Participants

Patient characteristics are provided in Table 1. Bern only included standardized patients from the University of Bern, as opposed to 10% of participants in Lausanne. Ten people were at the evening information in 2018 February, and after the first meeting one person declined further participation., as opposed to 10% of members in Lausanne. When Lausanne participants were asked their preferred means evaluating materials, 57% preferred questionnaires at home, 17% preferred in-person meetings, 3 9% were willing to do either, and 6% had no preference.

### Lausanne data collection and improvements to DAs

**Fig. 2:**
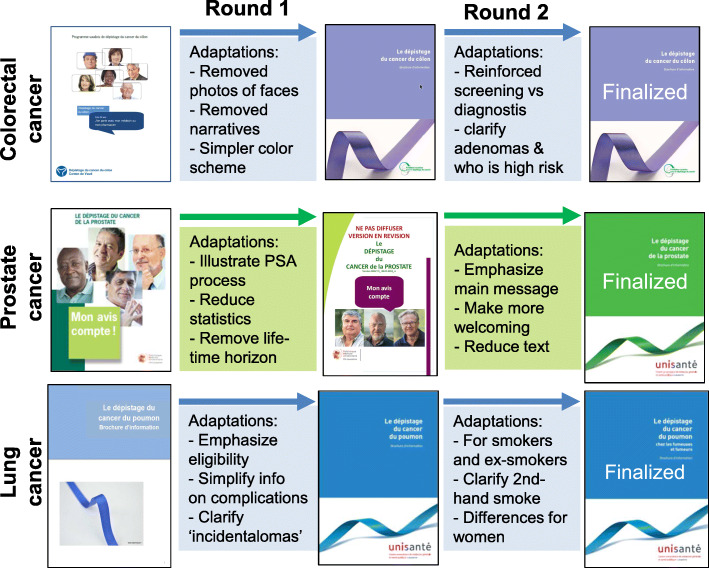
Changes made to Lausanne decision aids based on feedback from citizen group

Numerous changes were made to the content, appearance, language, and tone of three DAs based on the feedback of participants (Fig. 2). Regarding participant satisfaction, 26% of Lausanne participants said they had an excellent impression of their role in the group and 74% a very good impression; none responded Bad or Very Bad. Overall retention was good in Lausanne; participation remained stable over time and only 2 participants over 2 years asked that they no longer be solicited.

#### Colorectal cancer (CRC) screening DA

The group first provided feedback on an existing CRC screening DA and mailed letters used by several Cantonal CRC screening programs [15]. These programs mail materials to all citizens aged 50 to 69 to invite them to complete a fecal occult blood test or colonoscopy. We collected information during two in-person meetings and with a mailed questionnaire. Ten of 12 people invited attended each meeting and 26 of 38 participants (68%) replied by mail. Because multiple screening programs used the DA, there were often constraints to the changes we could make, which sometimes frustrated participants. Citizens helped clarify messages about adenomas and the meaning of high-risk criteria that can make people ineligible for the screening program. The mailed questionnaires generally showed high levels of knowledge and preparedness for making a decision about screening, but 62% of participants thought screening was only in case of symptoms of CRC. We therefore added emphasis to screening definitions.

#### Prostate cancer screening DA

The group then provided feedback on a prostate cancer screening DA [16], with 4 of 10 and 8 of 9 men attending two meetings and 22 of 38 people responding to a mailed questionnaire (58%). Main messages of the brochure were clarified, explaining that prostate cancer is controversial and not supported by all professional organizations [16].

#### Lung cancer screening DA

Finally, the group provided feedback on a new lung cancer screening DA, with 9 of 12 people attending a meeting and 24 of 38 people responding to a mailed questionnaire (63%). The structure of the brochure was adapted to emphasize smoking cessation and a section explaining additional tests after a positive scan, such as bronchoscopy, was shortened substantially.

### Bern data collection and improvements to materials

#### Feedback on information materials

**Fig. 3:**
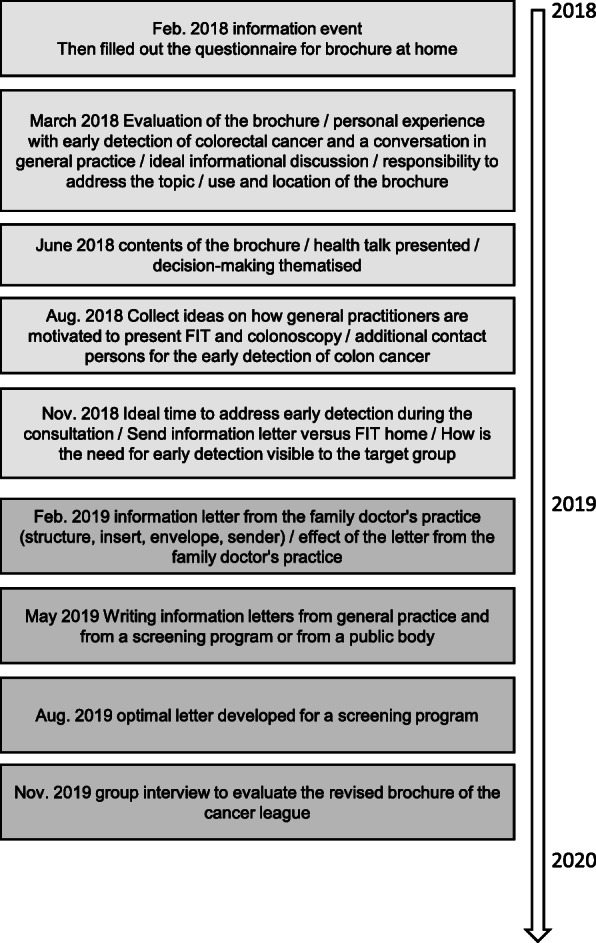
Subjects discussed at meetings in Bern

In the citizen group, work was carried out both in the large group, in smaller groups and individually on specific topics for the early detection of colon cancer (Fig. 3). The group was able to provide feedback twice on the brochure on colorectal cancer screening. In the first brochure and in the current brochure on colorectal cancer screening of the Swiss Cancer Screening, important findings and details from the Bernese support group were incorporated. Furthermore, the group developed two information letters in several sessions, one suitable for being sent out of a family doctor’s office and the other with the sender of a screening program, the latter was provided the screening programs. When developing these documents, particular emphasis was placed on simple, easy-to-understand language and clear illustrations. In addition, the participants dealt with the optimal time for the preventive consultation in a general practitioner consultation as well as other possible contacts regarding the early detection of colorectal cancer. Another finding out of the work with the group was the effect of words. The choice of words can have an unsettling or halting effect, which is particularly important in invitation letter from organized screening programs, in brochures and in discussions between doctors and patients. Between seven and ten participants were present at all meetings, with no drop-outs from the study.

#### Feedback on overall approach to CRC screening

One key insight from the Bern group was that the citizens felt they were able to decide whether to be screened for CRC screening and with which method on their own, based on decision aids and information documents received at home. This feedback was key to researchers who had set up their research project on targeting CRC screening decisions occurring in primary health care. This perspective taught the researchers humility and a wider perspective from persons invited to CRC screening. As researchers we knew general tendencies from the scientific literature, but were reminded that individual end-users know best what information they need and in what format to make a satisfactory decision about screening. Group members also mentioned they wanted better coordination between initiatives on the state-level, among health professionals such as pharmacists, gastroenterologists and primary care physicians on information documents and messages with regards to CRC screening. The research group, composed of primary care physicians, were reminded of the need to consider the wider healthcare system perspective for CRC screening decisions in the general population. Based on this insight, researchers more actively sought contact to network with further stakeholders to disseminate their findings and insights and focus on interprofessional collaboration. The group meetings enabled researchers to learn what matters to persons invited for CRC screening. They also experienced the wide range of opinions and perspectives from group members and that there is no solution working for all.

## Discussion

This article describes the experience of two Swiss centers for primary care forming a pool of end-users to provide repeated feedback on patient DAs. During iterative improvement cycles, the citizen-advisors provided valuable criticism that made materials and research projects evolve. Participants at in-person meetings became familiar with the concepts of SDM and provided higher-level observations over time. Due to local circumstances, there were important differences in the methodology used in Lausanne and Bern; these contrasting experiences can provide helpful insights for other DA developers.

Our citizen-advisors commented on multiple versions of our DAs, allowing both study groups to fulfill requirements in international DA guidelines for end-user feedback [6] without multiple cycles of recruitment to focus groups. Going forward, the Lausanne group also intends to pay citizen-advisors and use formal contracts, as was done in Bern, to ensure fair compensation. Repeated meetings with the same participants were helpful for both groups, though differences in methodology allowed us to attain different goals. In Lausanne, citizen-advisors were able to provide feedback more efficiently on subsequent DAs, such that fewer in-person meetings were required. However, our methodology did not allow us to measure informed choice among participants, as originally intended. In Bern, accumulated knowledge about the trial within which the group was nested [12] allowed citizen-advisors to provide critical feedback on our overall approach to CRC screening. Other DA developers should value experience gained by participants, which in our opinion outweighs the value of participants seeing a DA for the first time. In future studies we hope to combine feedback from citizen-advisors with one-to-one, ‘think-aloud’ interviews with persons from low-literacy groups. This approach will test comprehension of DAs developed with our current approach.

Two innovative points in our methodology should be highlighted. First, both study sites combined elements of community-based participatory research (CBPR) and quality improvement to develop a pragmatic means of involving end users in the creation of DAs. Projects using CBPR often employ community advisory boards (CABs) as a means of integrating stakeholders in key decisions and knowing local, context-specific knowledge [17]. We did not involve participants in strategic protocol decisions (aside from our dissemination plan), in the selection of topics to discuss, or explicitly in co-design [18]. Similar to CABs, we recruited from the population concerned by cancer screening, asked participants to serve as ‘representatives’ of all end-users, and maintained the same group of participants for in-person meetings to allow them to gain confidence and develop expertise. We also used elements of plan-do-study-act (PDSA) cycles, a methodology from quality improvement used to provide a structure for iterative testing of changes [11]. PDSA cycles aim to adapt complex interventions for local implementation. Though we based our initial DAs on examples in the international literature, we needed PDSA cycles to make the adaptations needed for successful dissemination in Switzerland.

Second, in both the Lausanne pilot phase and the Bern study phase, we recruited among standardized patients from our medical schools. In our experience, they have a heightened interest in potential improvements to the local medical system. Standardized patients can provide an easily-identifiable pool of participants, especially for topics like cancer screening without identifiable patient organizations. Standardized patients have been used previously in research projects as unannounced patients to measure care quality [19], but not to our knowledge for CBPR. In Lausanne, we also recruited from community organizations, which also identified people who can readily identify and discuss improvements to communication materials. Broadening to other sources of participants did seem to diversity the group, in having more members working full time in more professions. One limitation of all of our recruitment sources is that despite varied levels of education attainment, nearly all of our members seemed to have high levels of health literacy, though their health literacy was not formally measured using a validated scale.

Our two study sites allocated resources differently: in Lausanne we developed questionnaires that were mailed to citizens not participating in in-person meetings, while in Bern all meetings were audio-recorded, transcribed and analyzed by one of the study authors (BM). Patterns in responses to knowledge questions in the mailed questionnaires and written feedback provided valuable information beyond what was collected during in-person meetings. Questionnaires also provided individual-level information not always captured in group discussions. The systemic analysis of meeting transcripts by the Bern group allowed them to avoid a possible perception bias by the researchers, which significantly supported the quality of the data.

Strengths of this study include its parallel implementation at two study sites, the accumulation of 3 years experiences, and patient involvement in the preparation of the manuscript. Weaknesses include our small sample sizes and lack of comparator group, limiting the precision of our quantitative results and our ability to conclusively demonstrate that we reached our objective to develop a more efficient method of developing DAs. Meetings in Lausanne were not audio recorded, potentially introducing bias in our interpretation of meeting information. Our citizen partners participated fully in DA development and the reporting of results, but not in the choice of topics or the initial study protocol. As we gain confidence and experience in this area, and have existing contacts with citizen partners, we hope to involve them as partners earlier and earlier in the research process. Repeated meetings with the same participants might bias their responses and make our DAs less understandable for wider audiences. Also, we were able to develop multiple DAs with the same members because cancer screening is widely applicable to the general population aged 50 to 75; DAs addressing specific diseases will need to recruit patients with specific experience. The participants in Bern consisted exclusively of standardized patients, who brought with them an affinity for medical issues. With this prior medical knowledge, the support group likely differ from the average end-users from the population. We have not yet performed validation studies for our DAs and communication materials to assess their impact on routine care. Finally, because both groups were formed to discuss cancer screening in two academic centers for primary care, it is difficult to know the generalizability of our results.

## Conclusions

Smaller research groups like ours often struggle to involve end-users during the development of DAs. We describe our experience creating and maintaining citizen advisory groups to give repeated feedback on DAs using principles of CBPR and PDSA cycles. We think this pragmatic approach led to dramatic improvements in our DAs at lower cost than other methods. Future research should study the use of citizen advisory groups in other settings, methods for maintaining our group over time despite gaps in project-based research funding, and the expansion of our membership to include citizens with low health literacy.

## Supplementary Information

**Additional file 1.** GRIPP2 Reporting Checklist

**Additional file 2.** French-language questionnaires used by Lausanne group

## Data Availability

Not applicable.

## Abbreviations

CBPR: Community-based participatory research
CRC: Colorectal Cancer
DA: Decision aids
PDSA: Plan-Do-Study-Act
SDM: Shared decision making
