# Supplementary material for: Citizen advisory groups for the creation and improvement of decision aids: experience from two Swiss centers for primary care
Source: Res Involv Engagem. 2021 Jun 5;7:37. doi: 10.1186/s40900-021-00283-0 (PMC8179076; doi:10.1186/s40900-021-00283-0)
Supplement: Supplementary file 1 — Additional file 1. GRIPP2 Reporting Checklist [file 40900_2021_283_MOESM1_ESM.docx]

| **Section and topic** | **Item** | **Reported on page No** |
| --- | --- | --- |
| 1: Aim | Report the aim of PPI in the study | Page 4 – study aims were to have public involvement |
| 2: Methods | Provide a clear description of the methods used for PPI in the study | Pages 4 to 6 – describe public involvement in study and manuscript preparation |
| 3: Study results | Outcomes—Report the results of PPI in the study, including both positive and negative outcomes | Pages 6 to 8 – gives results of public involvement |
| 4: Discussion and conclusions | Outcomes—Comment on the extent to which PPI influenced the study overall. Describe positive and negative effects | Pages 8 to 10 |
| 5: Reflections/critical perspective | Comment critically on the study, reflecting on the things that went well and those that did not, so others can learn from this experience | Pages 10 to 11, we comment on study strengths and weaknesses |

1. *PPI* patient and public involvement
