# Supplementary material for: Citizen advisory groups for the creation and improvement of decision aids: experience from two Swiss centers for primary care
Source: Res Involv Engagem. 2021 Jun 5;7:37. doi: 10.1186/s40900-021-00283-0 (PMC8179076; doi:10.1186/s40900-021-00283-0)
Supplement: Supplementary file 2 — Additional file 2. French-language questionnaires used by Lausanne group [file 40900_2021_283_MOESM2_ESM.pdf]

Questionnaires used with Lausanne Citizen-advisory group

Colorectal cancer screening: pages 1 – 16

Prostate cancer screening: pages 17 – 31

Lung cancer screening: pages 32 - 42

Policlinique Médicale Universitaire CH-Lausanne

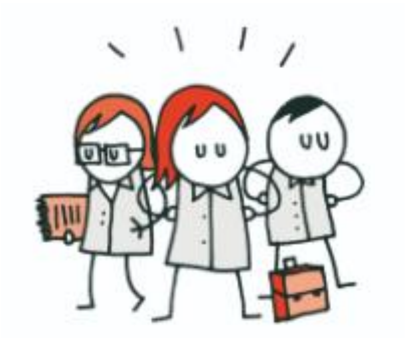

Votre avis compte…

La Policlinique médicale universitaire (PMU) implique les citoyen·ne·s afin d'améliorer la qualité de la documentation sur le dépistage des cancers, en collaboration avec la Fédération romande des consommateurs (FRC), Pro Senectute Vaud, la Faculté de biologie et de médecine (FBM) de l'UNIL, avec le soutien financier du Fonds de développement de la prévention et de la promotion de la santé du Service de la santé publique du canton de Vaud.

Nous souhaitons que la brochure «Le dépistage du cancer du côlon. Brochure d'information» soit compréhensible et soutienne les citoyen·ne·s dans leur prise de décision par rapport au dépistage du cancer du côlon.

Dans ce but, nous vous invitons à remplir les questionnaires du présent document :

- Le questionnaire 1 vise à évaluer la qualité de la brochure et son utilité dans la prise de décision informée des citoyen·ne·s.
- Le questionnaire 2 aborde les modes de distribution de l'information et de la brochure.
- La dernière page « Conclusion – votre avis général », au dos de la brochure, vous permet de donner votre avis sur la démarche du projet.

En remplissant et nous retournant ces questionnaires, vous acceptez que les données soient utilisées de manière anonyme à des fins de recherche, de développement de brochures et de leur distribution aux citoyennes et citoyens. La participation est bénévole.

Nous vous serions reconnaissants de retourner ce document à l'aide de l'enveloppe jointe à notre courrier à :

Regula Cardinaux - Policlinique médicale universitaire - Rue du Bugnon 44 - 1011 Lausanne
regula.cardinaux@hospvd.ch – 021/314.20.14

Evaluation de la brochure :
« Le dépistage du cancer du côlon. Brochure d’information »

Afin de nous aider à améliorer la brochure « Le dépistage du cancer du côlon. Brochure d’information » **nous aimerions que vous répondiez attentivement aux questions ci-dessous en cochant les cases appropriées (une réponse par question) et en remplissant par un texte lorsque cela vous est proposé. Certaines questions se ressemblent, car elles concernent les deux tests de dépistage du cancer du côlon, la détection de sang occulte dans les selles et la coloscopie.**
Vous pouvez bien sûr vous aider de la brochure qui ne doit pas être diffusée, car en cours d'élaboration.

Dans le présent document, les termes employés pour désigner des personnes sont pris au sens générique; ils ont à la fois valeur d’un féminin et d’un masculin.

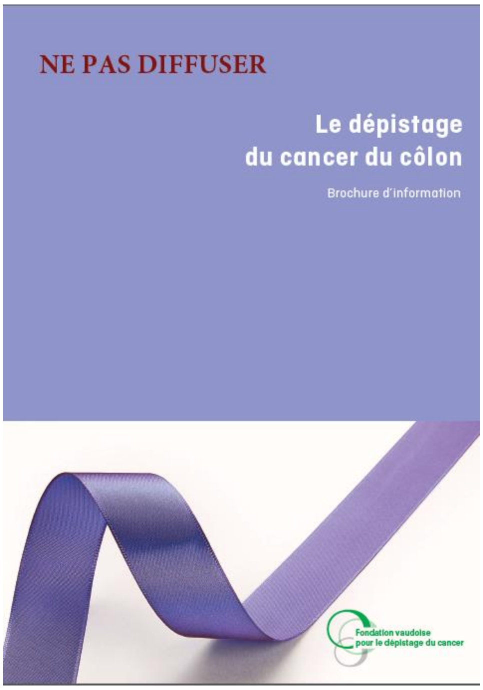

CONNAISSANCES

1. Pour quelle personne un test de dépistage du cancer du côlon est-il recommandé?

☐ Une personne en bonne santé

☐ Une personne avec des douleurs abdominales ou du sang dans les selles (une personne qui a des symptômes de cancer du côlon)

☐ *Je ne sais pas/ Je ne souhaite pas répondre*

2. Pour une personne dans la population générale*, à partir de quel âge est-il recommandé d'effectuer régulièrement un test de dépistage du cancer du côlon ?

☐ 30 ans ☐ 40 ans ☐ 50 ans ☐ 60 ans ☐ *Je ne sais pas*

** personne qui n'a aucun membre de la famille (père, mère, frère ou sœur) qui a eu un cancer du côlon ou des polypes*

3. Si un résultat de test est anormal, cela signifie-t-il toujours que la personne a un cancer du côlon ?

☐ Oui ☐ Non ☐ *Je ne sais pas*

4. Quel est le risque d'une complication sévère (saignement nécessitant une hospitalisation ou perforation du colon) lors d'une coloscopie ?

Très fréquent *(+ de 1/10)*	***Fréquent*** *(+ de 1/100 et - de 1/10)*	***Occasionnel*** *(+ de 1/ 1'000 et - de 1/100)*	***Rare*** *(+ de 1/10'000 et - de 1/1'000)*	***Très rare*** *(- de 1/10'000)*	***Je ne sais pas***
☐	☐	☐	☐	☐	☐

5. Pour une personne dans la population générale, quel test de dépistage est-il recommandé de faire une fois tous les deux ans ?

☐ Recherche de sang occulte dans les selles ☐ Coloscopie

☐ Aucun ☐ *Je ne sais pas*

6. Pour une personne dans la population générale, quel test de dépistage est-il recommandé de faire une fois tous les dix ans ?

☐ Recherche de sang occulte dans les selles ☐ Coloscopie

☐ Aucun ☐ *Je ne sais pas*

7. Quel test de dépistage permet de détecter le plus de polypes avant qu'ils n'évoluent en cancer du côlon ?

☐ Recherche de sang occulte dans les selles ☐ Coloscopie

☐ Aucun ☐ *Je ne sais pas*

8. Si un résultat de test est normal, est-il possible que la personne ait tout de même un cancer du côlon ?

☐ Oui ☐ Non ☐ *Je ne sais pas*

9. Sur 100 personnes qui *ne font PAS* de test de dépistage, environ combien de personnes mourront d'un cancer du côlon avant l'âge de 80 ans ?

☐ Aucune ☐ 1 personne ☐ 2 personnes ☐ 10 personnes ☐ 30 personnes
☐ 50 personnes ☐ 100 personnes ☐ *Je ne sais pas*

10. Sur 100 personnes qui *font un test* de dépistage régulier, environ combien de personnes mourront d'un cancer du côlon avant l'âge de 80 ans ?

☐ Aucune ☐ 1 personne ☐ 2 personnes ☐ 10 personnes ☐ 30 personnes
☐ 50 personnes ☐ 100 personnes ☐ *Je ne sais pas*

ATTITUDE et INTENTION

11. Sur une échelle de 1 à 5 *(1 étant le plus négatif et 5 le plus positif)*, que pensez-vous de chacune des six déclarations ci-dessous sur le dépistage du cancer du côlon ?

Pour moi, faire le dépistage du cancer du côlon …

	1	2	3	4	5	
**11a.** …est une mauvaise chose	○	○	○	○	○	…n'est pas une mauvaise chose
**11b.** …n'est pas bénéfique	○	○	○	○	○	…est bénéfique
**11c.** …a des inconvénients	○	○	○	○	○	…n'a pas d'inconvénients
**11d.** …n'est pas une bonne chose	○	○	○	○	○	…est une bonne chose
**11e.** …n'a pas d'avantages	○	○	○	○	○	…a des avantages
**11f.** …n'est pas important	○	○	○	○	○	…est important

12. Mes intentions par rapport au dépistage du cancer du côlon :

☐ J'ai la ferme intention d'effectuer un dépistage du cancer du côlon
☐ Je vais probablement effectuer un dépistage du cancer du côlon
☐ Je ne suis pas sûr d'effectuer un dépistage du cancer du côlon
☐ Je ne vais probablement pas effectuer un dépistage du cancer du côlon
☐ J'ai la ferme intention de ne pas effectuer un dépistage du cancer du côlon
☐ *Je ne sais pas/Je ne souhaite pas répondre*

13. Quelle option de dépistage préférez-vous après la lecture de la brochure ? Si je choisissais de faire le dépistage du cancer du côlon, je ferais une…

☐ Recherche de sang occulte dans les selles

☐ Coloscopie

☐ *Je ne sais pas/Je ne souhaite pas répondre*

a. Si vous avez répondu « la recherche de sang occulte dans les selles », quel est votre niveau d'intention de faire ce test :

☐ J'ai la ferme intention d'effectuer une recherche de sang occulte dans les selles

☐ Je vais probablement effectuer une recherche de sang occulte dans les selles

☐ Je ne suis pas sûr d'effectuer une recherche de sang occulte dans les selles

☐ Je ne vais probablement pas effectuer une recherche de sang occulte dans les selles

☐ J'ai la ferme intention de ne pas effectuer une recherche de sang occulte dans les selles

☐ *Je ne sais pas/Je ne souhaite pas répondre*

b. Si vous avez répondu « coloscopie », quel est votre niveau d'intention de faire ce test :

☐ J'ai la ferme intention d'effectuer une coloscopie

☐ Je vais probablement effectuer une coloscopie

☐ Je ne suis pas sûr d'effectuer une coloscopie

☐ Je ne vais probablement pas effectuer une coloscopie

☐ J'ai la ferme intention de ne pas effectuer une coloscopie

☐ Je ne sais pas/Je ne souhaite pas répondre

		Oui	Non
**S**ûr	Etes-vous certain de ce qui constitue le meilleur choix pour vous ?	☐	☐
**U**tilité de l'information	Est-ce que vous connaissez les bénéfices et risques de chacune des options	☐	☐
**R**isque-bénéfices à balancer	Avez-vous le sentiment de savoir ce qui est le plus important pour vous à l'égard des risques et bénéfices ?	☐	☐
**E**ncouragement	Avez-vous suffisamment de soutien pour faire votre choix ?	☐	☐

The SURE Test © Connor and Légaré, 2008

INQUIETUDE ET REGRET

14. A quel point êtes-vous inquiet à l'idée de pouvoir avoir un cancer du côlon ?

Pas du tout inquiet	*Un peu inquiet*	*Assez inquiet*	*Très inquiet*	*Je ne sais pas/Je ne souhaite pas répondre*
☐	☐	☐	☐	☐

15. A quel degré approuvez-vous l'affirmation suivante :

a. « Il est possible que plus tard je regrette d'avoir fait un dépistage » ?

☐ J'approuve fortement ☐ J'approuve ☐ Je ne peux me prononcer

☐ Je n'approuve pas ☐ Je n'approuve pas du tout

☐ *Je ne sais pas/Je ne souhaite pas répondre*

b. « Il est possible que plus tard je regrette de <u>ne pas</u> avoir fait un dépistage » ?

☐ J'approuve fortement ☐ J'approuve ☐ Je ne peux me prononcer

☐ Je n'approuve pas ☐ Je n'approuve pas du tout

☐ *Je ne sais pas/Je ne souhaite pas répondre*

EVALUATION DE LA BROCHURE D'INFORMATION

16. Avez-vous lu la brochure « Le dépistage du cancer du côlon» ?

☐ En partie ☐ Au complet ☐ *Je ne souhaite pas répondre*

17. Avez-vous discuté du contenu de la brochure avec un professionnel de la santé ?

☐ Non ☐ Oui, avec un médecin ☐ Oui, avec un pharmacien.ne

☐ Oui, avec un autre professionnel de la santé ☐ *Je ne souhaite pas répondre*

18. Environ combien de temps avez-vous pris pour lire la brochure « Le dépistage du cancer du côlon» ?

.... minutes

19. A quel point les informations contenues dans la brochure étaient-elles nouvelles pour vous ?

☐ Aucune information nouvelle ☐ Peu d'informations nouvelles

☐ Beaucoup d'informations nouvelles ☐ Entièrement nouvelles

☐ *Je ne sais pas/Je ne souhaite pas répondre*

20. Comment caractérisez-vous la taille de la brochure qui était nouvelle pour vous ?

☐ Vraiment trop longue ☐ Un peu trop longue ☐ Juste bien

☐ Un peu trop courte ☐ Vraiment trop courte

☐ *Je ne sais pas/Je ne souhaite pas répondre*

21. A quel point avez-vous trouvé la brochure neutre par rapport…

a. …au dépistage ?

☐ Clairement en faveur du dépistage ☐ Un peu en faveur du dépistage
☐ Complètement neutre ☐ Un peu en défaveur du dépistage
☐ Clairement en défaveur du dépistage ☐ *Je ne sais pas/Je ne souhaite pas répondre*

b. …au test de recherche de sang occulte dans les selles et de la coloscopie ?

☐ Clairement en faveur du test de recherche de sang occulte dans les selles
☐ Clairement en faveur de la coloscopie
☐ Un peu en faveur du test de recherche de sang occulte dans les selles
☐ Un peu en faveur de la coloscopie
☐ Complètement neutre
☐ Un peu en défaveur du test de recherche de sang occulte dans les selles
☐ Un peu en défaveur de la coloscopie
☐ Clairement en défaveur du test de recherche de sang occulte dans les selles
☐ Clairement en défaveur de la coloscopie
☐ *Je ne sais pas/Je ne souhaite pas répondre*

22. A quel degré approuvez-vous l'affirmation suivante :
« J'ai trouvé l'information de la brochure claire et simple à comprendre » ?

☐ J'approuve fortement ☐ J'approuve
☐ Je ne peux me prononcer ☐ Je n'approuve pas
☐ Je n'approuve pas du tout ☐ *Je ne sais pas/Je ne souhaite pas répondre*

23. A quel degré approuvez-vous l'affirmation suivante :
« J'ai trouvé la brochure utile pour prendre ma décision par rapport au dépistage du cancer du côlon »

☐ J'approuve fortement ☐ J'approuve
☐ Je ne peux me prononcer ☐ Je n'approuve pas
☐ Je n'approuve pas du tout ☐ *Je ne sais pas/Je ne souhaite pas répondre*

24. A quel degré approuvez-vous l'affirmation suivante :
« Je recommanderais cette brochure à des personnes qui réfléchissent au dépistage du cancer du côlon » ?

☐ J'approuve fortement ☐ J'approuve
☐ Je ne peux me prononcer ☐ Je n'approuve pas
☐ Je n'approuve pas du tout ☐ *Je ne sais pas/Je ne souhaite pas répondre*

25. A quel degré approuvez-vous l'affirmation suivante :
« Je fais confiance aux informations présentées dans la brochure » ?

☐ J'approuve fortement ☐ J'approuve
☐ Je ne peux me prononcer ☐ Je n'approuve pas
☐ Je n'approuve pas du tout ☐ *Je ne sais pas/Je ne souhaite pas répondre*

26. Ajouter une page à la brochure sur les facteurs de risques et la prévention des cancers en général serait :

☐ Très utile ☐ Utile ☐ Je ne peux me prononcer ☐ Pas utile
☐ Pas du tout utile ☐ *Je ne sais pas/ Je ne souhaite pas répondre*

DONNEES PERSONNELLES

27. Quel est votre année de naissance ?

19 .... ☐ *Je ne souhaite pas répondre*

28. Etes-vous ?

☐ Un homme ☐ Une femme ☐ Autre .... ☐ *Je ne souhaite pas répondre*

29. Quel est votre pays d'origine ?

...

30. Avez-vous déjà fait un examen ou test de recherche de sang occulte dans les selles, c'est-à-dire un prélèvement des selles sur un papier de test spécial pour y détecter la présence de traces de sang invisible à l'œil nu ?

☐ Oui ☐ Non ☐ *Je ne souhaite pas répondre*

a. Si oui, quand avez-vous effectué ce test de recherche de sang occulte dans les selles pour la dernière fois ?

Mois .... Année ....

b. Était-ce au cours des trois derniers mois ?

☐ Oui ☐ Non ☐ *Je ne sais pas/ Je ne souhaite pas répondre*

c. Si oui, la dernière fois, pour quelle raison avez-vous effectué ce test de recherche de sang occulte dans les selles. Était-ce :

☐ À titre préventif ou de « check up », sans troubles ou symptômes auparavant
☐ À titre de contrôle suite à des douleurs ou à des symptômes
☐ À titre de contrôle suite à un test précédent qui avait révélé quelque chose
Autre raison : ....
☐ *Je ne sais pas/Je ne souhaite pas répondre*

31. Avez-vous déjà fait une coloscopie, c'est-à-dire un examen visuel du gros intestin par une fine sonde ?

☐ Oui ☐ Non ☐ *Je ne souhaite pas répondre*

Si oui, quand cette coloscopie a-t-elle été faite sur vous pour la dernière fois ?

Mois .... Année ....

a. Était-ce au cours des trois derniers mois ?

☐ Oui ☐ Non ☐ *Je ne sais pas/ Je ne souhaite pas répondre*

b. Si oui, la dernière fois, pour quelle raison avez-vous effectué cette coloscopie ? Était-ce :

☐ À titre préventif ou de « check up », sans trouble ou symptôme auparavant

☐ À titre de contrôle suite à des douleurs ou à des symptômes

☐ À titre de contrôle suite à un examen précédent qui avait révélé quelque chose

☐ Autre raison : ....

☐ *Je ne souhaite pas répondre*

32. Avez-vous un médecin de famille personnel ? C'est-à-dire un médecin chez lequel vous pouvez vous rendre pour la plupart de vos problèmes de santé ?

☐ Oui ☐ Non ☐ *Je ne souhaite pas répondre*

a. Si oui, au cours des douze derniers mois, combien de fois vous êtes-vous rendu chez votre médecin de famille (médecin généraliste) ? Si vous ne savez pas exactement, estimez

Nombre de fois : ....

☐ *Je ne sais pas/Je ne souhaite pas répondre*

33. Comment est votre état de santé ?

☐ Très bon ☐ Bon ☐ Assez bon ☐ Mauvais ☐ Très mauvais

☐ *Je ne sais pas/ Je ne souhaite pas répondre*

34. Quel est le plus haut degré de formation que vous avez achevée :

☐ Scolarité obligatoire ☐ Ecole de métier / apprentissage

☐ Maturité/baccalauréat ☐ Ecole professionnelle supérieure

☐ Haute école spécialisée/ pédagogique ☐Université

☐ Autre: ....

☐ Aucune ☐ *Je ne souhaite pas répondre*

35. Quel est le métier exercé ?...

36. J'ai eu connaissance du projet par :

☐ Fédération romande des consommateurs ☐ Pro Senectute Vaud

☐ Université de Lausanne – patients simulés

☐ Policlinique médicale universitaire (PMU)

☐ Autre : ....

Transmission et distribution de l’information et de la brochure sur le dépistage du cancer du côlon

Afin de nous aider à définir le mode distribution de la brochure « Le dépistage du cancer du côlon. Brochure d’information», **nous aimerions que vous répondiez attentivement aux questions ci-dessous en cochant les cases appropriées et en remplissant par un texte lorsque cela vous est proposé. Pour certaines questions, vous pouvez cocher plusieurs réponses.**

Dans le présent document, les termes employés pour désigner des personnes sont pris au sens générique; ils ont à la fois valeur d’un féminin et d’un masculin.

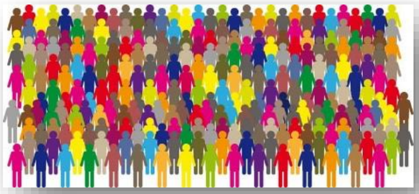

Questionnaire 2

1. AVANT la participation à ce projet :

1a. Aviez-vous déjà des informations sur le cancer du côlon avant votre participation à ce projet ?

☐ Oui ☐ Non ☐ *Je ne souhaite pas répondre*

1b. Aviez-vous déjà des informations sur le <u>dépistage</u> du cancer du côlon ?

☐ Oui ☐ Non ☐ *Je ne souhaite pas répondre*

1c. Si vous avez répondu oui à 1b, comment avez-eu connaissance du cancer du côlon et/ou de son dépistage ? (plusieurs réponses possibles)

☐ Par des amis/connaissances ou membres de ma famille ☐ Par mon médecin

☐ Par mon pharmacien ☐ Par d'autres professionnels de la santé

☐ Par une documentation médicale :

☐ dans la salle d'attente du médecin/de l'hôpital

☐ à la pharmacie

☐ autre : ....

☐ Par de l'information reçue à domicile :

☐ par poste

☐ par e-mail

☐ par téléphone

☐ Par des articles de journaux ou de magazines ☐ Par des affiches

☐ Par internet : ☐ par des sites web

☐ par des réseaux sociaux

☐ Par la radio

☐ Par la télévision

☐ Par le Programme cantonal de dépistage du cancer du côlon

☐ Autre : ....

☐ *Je ne sais pas/Je ne souhaite pas répondre*

… à quel point l'information a-t-elle été facile d'accès (1 = le plus négatif / 10 = le plus positif) ?

…pas facile du tout 1 ◯ 2 ◯ 3 ◯ 4 ◯ 5 ◯ 6 ◯ 7 ◯ 8 ◯ 9 ◯ 10 ◯ …très facile

☐ *Je ne sais pas/Je ne souhaite pas répondre*

… à quel point l'information trouvée a-t-elle répondu à vos besoins (1 = le plus négatif / 10 = le plus positif) :

…pas du tout 1 ◯ 2 ◯ 3 ◯ 4 ◯ 5 ◯ 6 ◯ 7 ◯ 8 ◯ 9 ◯ 10 ◯ …complètement

☐ *Je ne sais pas/Je ne souhaite pas répondre*

2. Interlocuteurs

2a. Sur une échelle de 1 (pas du tout approprié) à 10 (complètement approprié), à quel point les interlocuteurs suivants sont-ils selon vous appropriés pour parler du dépistage du cancer du côlon avec vous : (plusieurs réponses possibles)

Médecin	1 ◯	2 ◯	3 ◯	4 ◯	5 ◯	6 ◯	7 ◯	8 ◯	9 ◯	10 ◯
Pharmacien	1 ◯	2 ◯	3 ◯	4 ◯	5 ◯	6 ◯	7 ◯	8 ◯	9 ◯	10 ◯
Assistante médicale	1 ◯	2 ◯	3 ◯	4 ◯	5 ◯	6 ◯	7 ◯	8 ◯	9 ◯	10 ◯
Infirmière	1 ◯	2 ◯	3 ◯	4 ◯	5 ◯	6 ◯	7 ◯	8 ◯	9 ◯	10 ◯
Autre professionnel de la santé	1 ◯	2 ◯	3 ◯	4 ◯	5 ◯	6 ◯	7 ◯	8 ◯	9 ◯	10 ◯
Ami/ Connaissance/ Famille	1 ◯	2 ◯	3 ◯	4 ◯	5 ◯	6 ◯	7 ◯	8 ◯	9 ◯	10 ◯
Autre	1 ◯	2 ◯	3 ◯	4 ◯	5 ◯	6 ◯	7 ◯	8 ◯	9 ◯	10 ◯

☐ *Je ne sais pas/ Je ne souhaite pas répondre*

2b. A quel degré approuvez-vous l'affirmation suivante :
« Je trouve nécessaire et utile de discuter avec mon médecin pour décider avec lui de faire le dépistage du cancer du côlon ou pas » ?

☐ J'approuve fortement ☐ J'approuve ☐ Je ne peux me prononcer
☐ Je n'approuve pas ☐ Je n'approuve pas du tout
☐ *Je ne sais pas/Je ne souhaite pas répondre*

3. Diffusion de l'information sur le dépistage du cancer du côlon

3a. Pensez-vous que les citoyennes et citoyens souhaiteraient recevoir de l'information sur le dépistage du cancer du côlon ?

☐ Oui ☐ Non ☐ *Je ne sais pas/Je ne souhaite pas répondre*

3b. Si vous avez répondu oui à 3a, quelle.s serai.en.t selon vous la/les meilleure.s approche.s pour transmettre l'information sur le dépistage du cancer du côlon aux citoyennes et citoyens ? (plusieurs réponses possibles)

Interlocuteur/distributeur :

☐ Médecin ☐ Pharmacien
☐ Autres professionnels (assistante médicale ou infirmier)
☐ Programme cantonal de dépistage du cancer du côlon
☐ Service de la Santé Publique cantonal
☐ Autre : .... ☐ *Je ne sais pas/Je ne souhaite pas répondre*

Support de distribution:

☐ Papier : ☐ brochure
☐ feuillet
☐ affiche
☐ autre : ....

☐ Electronique : ☐ brochure
☐ feuillet
☐ application
☐ autre : ....

☐ Autre : .... ☐ *Je ne sais pas/Je ne souhaite pas répondre*

Mode de distribution :

☐ Salle(s) d'attente du médecin/de l'hôpital ☐ A la pharmacie
☐ Information transmise à domicile : ☐ par poste
☐ par E-mail
☐ par téléphone
☐ Médias : ☐ Articles de journaux ou de magazines
☐ Radio
☐ Télévision
☐ Par internet : ☐ Sites web ☐ Réseaux sociaux
☐ Autre : .... ☐ *Je ne sais pas/Je ne souhaite pas répondre*

4. Diffusion de la brochure sur le dépistage du cancer du côlon.

4a. Que pensez-vous du fait de diffuser la brochure sur le dépistage du cancer du côlon aux citoyennes et citoyens entre 50 ans et 69 ans ?

☐ Toujours souhaitable ☐ Parfois souhaitable
☐ Je ne peux me prononcer ☐ Rarement souhaitable
☐ Jamais souhaitable ☐ *Je ne sais pas/Je ne souhaite pas répondre*

4b. Si votre réponse est positive à 4a, quelle.s serai.en.t selon vous la/les meilleure.s approche(s) pour faire connaître la brochure sur le dépistage du cancer du côlon aux citoyennes et citoyens ? (plusieurs réponses possibles)

Interlocuteur/distributeur :

☐ Distribution par le médecin ☐ Distribution par le pharmacien
☐ Distribution par d'autres professionnels (assistante médicale, infirmier, etc.)
☐ Distribution par le Programme cantonal de dépistage du cancer du côlon
☐ Distribution par le Service de la Santé Publique cantonal

☐ Autre : .... ☐ *Je ne sais pas/Je ne souhaite pas répondre*

Support de distribution de la brochure:

☐ Version papier ☐ Version électronique

☐ Autre : .... ☐ *Je ne sais pas/Je ne souhaite pas répondre*

Mode de distribution de la brochure:

☐ Mise à disposition dans les salles d'attente du médecin/de l'hôpital

☐ Mise à disposition à la pharmacie

☐ Brochure envoyée à domicile : ☐ par poste

☐ par email

☐ Mise à disposition sur internet : ☐ sites web

☐ réseaux sociaux

☐ Autre : .... ☐ *Je ne sais pas/Je ne souhaite pas répondre*

Commentaires :

....

....

....

....

....

....

....

....

Merci de donner votre avis sur la démarche au dos du document

Conclusion – votre avis général

1. **Que pensez-vous du nombre de questions soumises en une fois ?**

☐ Beaucoup trop nombreuses ☐ Trop nombreuses
☐ Juste bien ☐ Pas assez nombreuses ☐ Pas d'avis

2. **Que pensez-vous du temps nécessaire pour répondre aux questionnaires?**

☐ Beaucoup trop long ☐ Trop long ☐ Juste bien
☐ Pas assez long ☐ Pas d'avis

3. **Quelle est votre impression générale de la démarche d'évaluation de la brochure par questionnaires à remplir à domicile?**

☐ Excellente ☐ Bonne ☐ Mauvaise
☐ Très mauvaise ☐ Pas d'avis

4. **Quelle est le meilleur support pour des questionnaires à remplir à domicile?**

☐ Papier ☐ Fichier électronique
☐ Questionnaire online ☐ Questions posées par téléphone
☐ Autre : .... ☐ Pas d'avis

5. **Comment avez-vous trouvé le format et la présentation des questionnaires (livret, taille des caractères d'écriture, etc.) ?**

☐ Excellent ☐ Très bien
☐ Bien ☐ Moyen
☐ Mauvais ☐ Très mauvais
☐ Pas d'avis

Points positifs/négatifs :

....

....

Suggestions :

....

....

....

Date : ....

Merci de votre participation active à nos efforts pour améliorer la transmission d'information sur le dépistage du cancer du côlon aux citoyennes et citoyens concerné.e.s !

Version septembre 2018

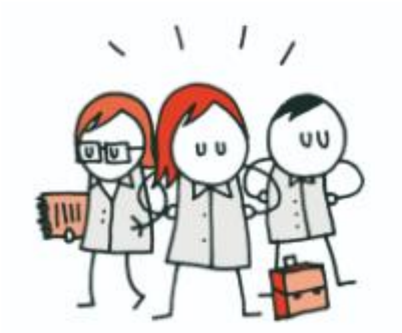

Votre avis compte…

Unisanté implique les citoyen·ne·s afin d'améliorer la qualité de la documentation sur le dépistage des cancers, en collaboration avec la Fédération romande des consommateurs (FRC), Pro Senectute Vaud, la Faculté de biologie et de médecine (FBM) de l'UNIL, avec le soutien financier du Fonds de développement de la prévention et de la promotion de la santé du Service de la santé publique du canton de Vaud.

Nous souhaitons que la brochure « Le dépistage du cancer de la prostate. Brochure d'information » soit compréhensible et soutienne les citoyens dans leur prise de décision par rapport au dépistage du cancer de la prostate.

Dans ce but, nous vous invitons à remplir les questionnaires du présent document :

- Le questionnaire 1 vise à évaluer la qualité de la brochure et son utilité dans la prise de décision informée des citoyens.
- Le questionnaire 2 aborde les modes de distribution de l'information et de la brochure.
- La dernière page « Conclusion – votre avis général », au dos de ce document, vous permet de donner votre avis sur la démarche du projet.

En remplissant et nous retournant ces questionnaires, vous acceptez que les données soient utilisées de manière anonyme à des fins de recherche, de développement de brochures et de leur distribution aux citoyennes et citoyens. La participation est bénévole.

Nous vous serions reconnaissants de retourner ce document à l'aide de l'enveloppe jointe à notre courrier à :

Regula Cardinaux – Unisanté - Rue du Bugnon 44 - 1011 Lausanne
regula.cardinaux@hospvd.ch – 021/314.20.14

Evaluation de la brochure :
« Le dépistage du cancer de la prostate : brochure d'information »

Afin de nous aider à améliorer la brochure « Le dépistage du cancer de la prostate : brochure d'information », **nous aimerions que vous répondiez attentivement aux questions ci-dessous en cochant les cases appropriées (une réponse par question) et en remplissant par un texte lorsque cela vous est proposé.**
Vous pouvez bien sûr vous aider de la brochure qui ne doit pas être diffusée, car en cours d'élaboration.
Si vous êtes une femme, nous vous demandons de répondre aux questions comme si vous deviez aviser ou prendre la décision avec un de vos proches. *Dans ce cas, certains termes employés pour désigner des personnes sont pris au sens générique; ils ont à la fois valeur d'un féminin et d'un masculin.*

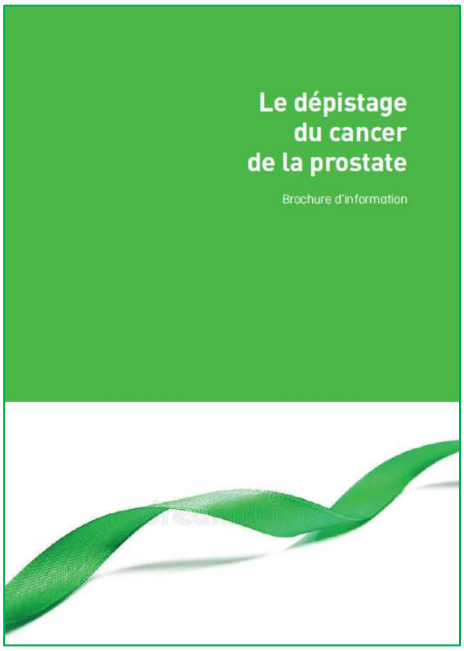

CONNAISSANCES

1. **A qui le médecin recommande-t-il un test de dépistage du cancer de la prostate?**
☐ à un homme en bonne santé ☐ à un homme avec symptômes de cancer
☐ à aucun homme, il n'y a pas de recommandation claire ☐ *Je ne sais pas*

2. **Pour un homme dans la population générale*, à partir de quel âge les médecins parlent-ils du test de dépistage du cancer de la prostate à leurs patients?**
☐ 30 ans ☐ 40 ans ☐ 50 ans ☐ 60 ans
☐ *Je ne sais pas*
* personne qui n'a aucun membre de la famille (père ou frère) qui a eu un cancer de la prostate

3. **Si un résultat de test PSA est anormal, cela signifie-t-il toujours que la personne a un cancer de la prostate?**
☐ Oui ☐ Non ☐ *Je ne sais pas*

4. **Pour un homme dans la population générale de plus de 50 ans, quelle est la fréquence des tests cités ci-dessous lors d'un dépistage ?**

	A chaque dépistage	Parfois	Que si nécessaire suite à un PSA anormal	Je ne sais pas
**Prise de sang, test PSA**	☐	☐	☐	☐
**Toucher rectal**	☐	☐	☐	☐
**Biopsie**	☐	☐	☐	☐

5. **Si un résultat de test PSA est normal, est-il possible que la personne ait tout de même un cancer de la prostate ?**
☐ Oui ☐ Non ☐ *Je ne sais pas*

6. **Sur 1000 personnes qui *font* un test de dépistage PSA régulièrement et dont on observe les conséquences durant 13 ans, environ combien d'hommes mourront d'un cancer de la prostate entre 55 et 70 ans ?**
☐ Aucun ☐ 1 homme ☐ 2 hommes ☐ 5 hommes
☐ 6 hommes ☐ 8 hommes ☐ 16 hommes ☐ 32 hommes
☐ *Je ne sais pas*

7. **Faire le dépistage augmente fortement la probabilité d'avoir une diagnostic de cancer de la prostate**
☐ Juste ☐ Faux ☐ *Je ne sais pas*

8. **Tous les cancers de la prostate entraîneront maladie et décès s'ils ne sont pas diagnostiqués et pas traités**

☐ Juste ☐ Faux ☐ *Je ne sais pas*

9. **Il est possible qu'un patient avec un cancer de la prostate qui n'entraînera pas d'atteinte à sa santé reçoive tout de même un traitement**

☐ Juste ☐ Faux ☐ *Je ne sais pas*

10. **Le dépistage peut mener des patients à prendre un traitement dont ils n'auraient pas besoin**

☐ Juste ☐ Faux ☐ *Je ne sais pas*

ATTITUDE ET INTENTION

11. **Que pensez-vous du test PSA ?**

Mettez une croix dans la case qui correspond à votre appréciation de chacune des sept affirmations du tableau ci-dessous :

A mon avis, faire le test PSA …

	1	2	3	4	5	
**11a**. …est une mauvaise chose	◯	◯	◯	◯	◯	…n'est pas une mauvaise chose
**11b**. …est bénéfique	◯	◯	◯	◯	◯	…n'est pas bénéfique
**11c.** …a des inconvénients	◯	◯	◯	◯	◯	…n'a pas d'inconvénients
**11d.** …est une bonne chose	◯	◯	◯	◯	◯	…n'est pas une bonne chose
**11e.** …a des avantages	◯	◯	◯	◯	◯	…n'a pas d'avantages
**11f.** …est important	◯	◯	◯	◯	◯	…n'est pas important

12. **Mes intentions par rapport au test PSA (choisir une réponse)**

Si vous êtes une femme, quelle recommendation auriez vous pour un proche?

☐ J'ai la ferme intention d'effectuer un dépistage par test PSA
☐ Je vais probablement effectuer un dépistage par test PSA
☐ Je ne suis pas sûr d'effectuer un dépistage par test PSA
☐ Je ne vais probablement pas effectuer un dépistage par test PSA
☐ J'ai la ferme intention de ne pas effectuer un dépistage par test PSA
☐ *Je ne sais pas/Je ne souhaite pas répondre*

INQUIETUDE ET REGRET

13. A quel point êtes-vous inquiet à l'idée de pouvoir avoir un cancer de la prostate ?
Si vous êtes une femme, quelle niveau d'inquiètude auriez vous pour un proche?
☐ Pas inquiet du tout ☐ Un peu inquiet ☐ Assez inquiet
☐ Très inquiet ☐ *Je ne souhaite pas répondre*

14. A quel degré approuvez-vous les deux affirmations suivantes :
Si vous êtes une femme, quel position par rapport aux affirmations suivantes auriez vous pour un proche ?

a. « Il est possible que plus tard je regrette d'avoir fait un dépistage » ?
☐ J'approuve fortement ☐ J'approuve ☐ Je ne peux me prononcer
☐ Je n'approuve pas ☐ Je n'approuve pas du tout
☐ *Je ne souhaite pas répondre*

b. « Il est possible que plus tard je regrette de ne pas avoir fait un dépistage » ?
☐ J'approuve fortement ☐ J'approuve ☐ Je ne peux me prononcer
☐ Je n'approuve pas ☐ Je n'approuve pas du tout
☐ *Je ne souhaite pas répondre*

EVALUATION DE LA BROCHURE D'INFORMATION

15. Avez-vous lu la brochure « Le dépistage du cancer de la prostate. Brochure d'infomation » …
☐ En partie ☐ Entièrement
☐ *Je ne sais pas/Je ne souhaite pas répondre*

16. Avez-vous discuté du contenu de la brochure avec un professionnel de la santé ?
☐ Non ☐ Oui, avec un médecin ☐ Oui, avec un pharmacien
☐ Oui, avec un autre professionnel de la santé
☐ *Je ne sais pas/Je ne souhaite pas répondre*

17. Environ combien de temps avez-vous pris pour lire la brochure ?
.... minutes

18. A quel point les informations contenues dans la brochure étaient-elles nouvelles pour vous ?
☐ Aucune nouvelle information ☐ Peu de nouvelles informations
☐ Beaucoup de nouvelles informations ☐ Entièrement nouvelles
☐ *Je ne sais pas/Je ne souhaite pas répondre*

19. Comment caractérisez-vous la taille de la brochure qui était nouvelle pour vous ?

☐ Vraiment trop longue ☐ Un peu trop longue
☐ Juste bien ☐ Un peu trop courte
☐ Vraiment trop courte ☐ *Je ne sais pas/Je ne souhaite pas répondre*

20. A quel point avez-vous trouvé la brochure neutre par rapport au dépistage ?

☐ Clairement en faveur du dépistage ☐ Un peu en faveur du dépistage
☐ Complètement neutre ☐ Un peu en défaveur du dépistage
☐ Clairement en défaveur du dépistage
☐ *Je ne sais pas/Je ne souhaite pas répondre*

21. A quel degré approuvez-vous l'affirmation suivante :
« J'ai trouvé l'information de la brochure claire et simple à comprendre » ?

☐ J'approuve fortement ☐ J'approuve ☐ Je ne peux me prononcer
☐ Je n'approuve pas ☐ Je n'approuve pas du tout
☐ *Je ne sais pas/Je ne souhaite pas répondre*

22. A quel degré approuvez-vous l'affirmation suivante :
« J'ai trouvé la brochure utile pour prendre une décision par rapport au dépistage du cancer de la prostate »
Si vous êtes une femme, est-ce que la brochure vous a préparé à soutenir un proche dans la décision de faire un dépisatge du cancer de la prostate ?

☐ J'approuve fortement ☐ J'approuve ☐ Je ne peux me prononcer
☐ Je n'approuve pas ☐ Je n'approuve pas du tout
☐ *Je ne sais pas/Je ne souhaite pas répondre*

23. A quel degré approuvez-vous l'affirmation suivante :
« Je recommanderais cette brochure à des personnes qui réfléchissent au dépistage du cancer de la prostate » ?

☐ J'approuve fortement ☐ J'approuve ☐ Je ne peux me prononcer
☐ Je n'approuve pas ☐ Je n'approuve pas du tout
☐ *Je ne sais pas/Je ne souhaite pas répondre*

24. A quel degré approuvez-vous l'affirmation suivante :
« Je fais confiance aux informations présentées dans la brochure » ?

☐ J'approuve fortement ☐ J'approuve ☐ Je ne peux me prononcer
☐ Je n'approuve pas ☐ Je n'approuve pas du tout
☐ *Je ne sais pas/Je ne souhaite pas répondre*

25. A quel degré approuvez-vous l'affirmation suivante :
« La brochure m'incite à aller discuter du dépistage du cancer de la prostate avec mon médecin et à prendre une décision avec lui à ce sujet » ?
Si vous êtes une femme : « La brochure m'incite à encourager un proche à aller discuter du dépistage du cancer de la prostate avec son médecin et à prendre une décision avec lui à ce sujet » ?

☐ J'approuve fortement ☐ J'approuve ☐ Je ne peux me prononcer
☐ Je n'approuve pas ☐ Je n'approuve pas du tout
☐ *Je ne sais pas/Je ne souhaite pas répondre*

CHOIX - OPTION

26. Concernant le dépistage du cancer de la prostate :

		Oui	Non
Sûr	Etes-vous certain de ce qui constitue le meilleur choix pour vous ?	☐	☐
Utilité de l'information	Est-ce que vous connaissez les bénéfices et risques de chacune des options	☐	☐
Risque-bénéfices à balancer	Avez-vous le sentiment de savoir ce qui est le plus important pour vous à l'égard des risques et bénéfices ?	☐	☐
Encouragement	Avez-vous suffisamment de soutien pour faire votre choix ?	☐	☐

The SURE Test © Connor and Légaré, 2008, avec adaptations pour le conjoint

27. Merci de décrire ci-dessous en quelques mots ce qui vous inciterait à aller voir votre médecin et à prendre une décision avec lui sur le dépistage du cancer de la prostate :
Si vous êtes une femme, qu'est-ce qui vous inciterait à encourager un proche à aller chez le médecin ?

....
....
....
....
....
....

☐ *Je ne sais pas/Je ne souhaite pas répondre*

28. Quelle option préférez-vous ?

Si vous êtes une femme, quelle option préconisez-vous pour un proche ?

☐ Pas de dépistage du cancer de la prostate

☐ Dépistage par test PSA

☐ Je ne suis pas certain de mon choix

☐ *Je ne sais pas/Je ne souhaite pas répondre*

DONNEES PERSONNELLES

29. Quel est votre année de naissance ?

19 .... ☐ *Je ne souhaite pas répondre*

30. Etes-vous ?

☐ Un homme ☐ Une femme ☐ Autre ....

☐ *Je ne souhaite pas répondre*

31. Quel est votre pays de naissance ?

....

32. Avez-vous déjà fait un test PSA, c'est-à-dire une prise de sang permettant de détecter la présence d'antigène spécifique de la prostate, protéine libérée dans le sang par la prostate ? Ne pas remplir si vous êtes une femme

☐ Oui ☐ Non ☐ *Je ne sais pas/Je ne souhaite pas répondre*

a. Si oui, quand avez-vous effectué ce test PSA pour la dernière fois ?

Mois :............. Année .... **Ne pas remplir si vous êtes une femme**

b. Si oui, la dernière fois, pour quelle raison avez-vous effectué ce test PSA, était-ce : Ne pas remplir si vous êtes une femme

☐ À titre préventif ou de « check up », sans troubles ou symptômes auparavant

☐ À titre de contrôle suite à des douleurs ou à des symptômes

☐ À titre de contrôle suite à un test précédent qui avait révélé quelque chose

☐ Autre raison : ....

☐ *Je ne sais pas/Je ne souhaite pas répondre*

33. Avez-vous déjà eu une biopsie de la prostate, c'est-à-dire un prélèvement de la prostate par aiguille dans le but de l'analyser au microscope?

Ne pas remplir si vous êtes une femme

☐ Oui ☐ Non ☐ *Je ne souhaite pas répondre*

a. **Si oui, quand cette biopsie a-t-elle été faite ?**
Mois :............. Année ....**Ne pas remplir si vous êtes une femme**

b. **Si oui, la dernière fois, pour quelle raison vous a-t-on proposé une biopsie de la prostate? Était-ce : Ne pas remplir si vous êtes une femme**
☐ À titre préventif ou de « check up », sans troubles ou symptômes auparavant
☐ À titre de contrôle suite à des douleurs ou à des symptômes
☐ À titre de contrôle suite à un examen précédent qui avait révélé quelque chose
☐ Autre raison : ....
☐ *Je ne souhaite pas répondre*

34. Avez-vous un médecin de famille personnel ? C'est-à-dire un médecin chez lequel vous pouvez vous rendre pour la plupart des problèmes de santé ?
☐ Oui ☐ Non ☐ *Je ne souhaite pas répondre*

a. **Si oui, au cours des douze derniers mois, combien de fois vous êtes-vous rendu chez votre médecin de famille (médecin généraliste) ? Si vous ne savez pas exactement, estimez :**
☐ Nombre de fois : .... ☐ *Je ne sais pas/Je ne souhaite pas répondre*

35. Comment est votre état de santé?
☐ Très bon ☐ Bon ☐ Assez bon
☐ Mauvais ☐ Très mauvais
☐ *Je ne sais pas/Je ne souhaite pas répondre*

36. Quel est le plus haut degré de formation que vous avez achevée :
☐ Scolarité obligatoire ☐ Ecole de métier / apprentissage
☐ Maturité/baccalauréat ☐ Ecole professionnelle supérieure
☐ Haute école spécialisée/ pédagogique ☐Université
☐ Autre: ....
☐ Aucune ☐ *Je ne souhaite pas répondre*

37. Quel est le métier exercé ? ....

38. J'ai eu connaissance du projet par :
☐ Fédération romande des consommateurs
☐ Pro Senectute Vaud
☐ Université de Lausanne – patients simulés
☐ Policlinique médicale universitaire (PMU)
☐ Autre :

Transmission et distribution de l'information et de la brochure sur le dépistage du cancer de la prostate

Afin de nous aider à définir le mode de distribution de la brochure « Le dépistage du cancer de la prostate. Brochure d'information», **nous aimerions que vous répondiez attentivement aux questions ci-dessous en cochant les cases appropriées et en remplissant par un texte lorsque cela vous est proposé. Pour certaines questions, vous pouvez cocher plusieurs réponses.**

Si vous êtes une femme, ces questions vous sont aussi adressées. *Dans ce cas, certains termes employés pour désigner des personnes sont pris au sens générique; ils ont à la fois valeur d'un féminin et d'un masculin.*

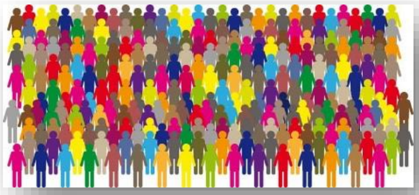

1. AVANT la participation à ce projet :

1a. Aviez-vous déjà des informations sur le cancer de la prostate ?

☐ Oui ☐ Non ☐ *Je ne souhaite pas répondre*

1b. Aviez-vous déjà des informations sur le dépistage du cancer de la prostate?

☐ Oui ☐ Non ☐ *Je ne souhaite pas répondre*

1c. Si vous avez répondu oui à 1b, comment avez-eu connaissance du cancer de la prostate et/ou de son dépistage ?

☐ Par des amis/connaissances ou membres de ma famille ☐ Par mon médecin

☐ Par mon pharmacien ☐ Par d'autres professionnels de la santé

☐ Par une documentation médicale :

- ☐ dans la salles d'attente du médecin/de l'hôpital
- ☐ à la pharmacie
- ☐ autre : ....

☐ Par de l'information reçue à domicile :

- ☐ par poste
- ☐ par e-mail
- ☐ par téléphone

☐ Par des médias

- ☐ Par des articles de journaux ou de magazines
- ☐ Par des affiches
- ☐ Par la radio
- ☐ Par la télévision
- ☐ Par internet : ☐ sites web ☐ réseaux sociaux

☐ Autre : ....

☐ *Je ne sais pas/ Je ne souhaite pas répondre*

… à quel point l'information a-t-elle été facile d'accès ?
(1 = le plus négatif / 10 = le plus positif) ?

…pas facile du tout	1	2	3	4	5	6	7	8	9	10	…très facile
	◯	◯	◯	◯	◯	◯	◯	◯	◯	◯	

☐ *Je ne sais pas/ Je ne souhaite pas répondre*

… à quel point l'information trouvée a-t-elle répondu à vos besoins :
(1 = le plus négatif / 10 = le plus positif) :

…pas du tout	1	2	3	4	5	6	7	8	9	10	…complètement
	◯	◯	◯	◯	◯	◯	◯	◯	◯	◯	

☐ *Je ne sais pas/ Je ne souhaite pas répondre*

2. Interlocuteurs

2a. Sur une échelle de 1 (pas du tout approprié) à 10 (complètement approprié), à quel point les interlocuteurs suivants sont-ils selon vous appropriés pour parler du dépistage du cancer de la prostate avec vous ou un proche:

	1	2	3	4	5	6	7	8	9	10
Médecin	◯	◯	◯	◯	◯	◯	◯	◯	◯	◯
Pharmacien	◯	◯	◯	◯	◯	◯	◯	◯	◯	◯
Assistante médicale	◯	◯	◯	◯	◯	◯	◯	◯	◯	◯
Infirmière	◯	◯	◯	◯	◯	◯	◯	◯	◯	◯
Autre professionnel de la santé	◯	◯	◯	◯	◯	◯	◯	◯	◯	◯
Ami/ Connaissance/ Famille	◯	◯	◯	◯	◯	◯	◯	◯	◯	◯
Autre	◯	◯	◯	◯	◯	◯	◯	◯	◯	◯

☐ *Je ne sais pas/Je ne souhaite pas répondre*

2b. A quel degré approuvez-vous l'affirmation suivante :
« Je trouve nécessaire et utile de discuter avec mon médecin pour décider avec lui de faire le dépistage du cancer de la prostate ou pas » ?
Si vous êtes une femme, à quelle degré l'aprouveriez-vous pour un proche ?

☐ J'approuve fortement ☐ J'approuve ☐ Je ne peux me prononcer
☐ Je n'approuve pas ☐ Je n'approuve pas du tout
☐ *Je ne sais pas/Je ne souhaite pas répondre*

3. Diffusion de l'information sur le dépistage du cancer de la prostate

3a. Pensez-vous que les citoyennes et citoyens souhaiteraient recevoir de l'information sur le dépistage du cancer de la prostate?

☐ Oui ☐ Non ☐ *Je ne sais pas/Je ne souhaite pas répondre*

3b. Si oui, quelle.s serai.en.t selon vous la/les meilleure.s approche.s pour transmettre l'information sur le dépistage du cancer de la prostate aux citoyennes et citoyens?

Interlocuteur/distributeur :

☐ Médecin ☐ Pharmacien
☐ Autres professionnels (assistante médicale ou infirmier)
☐ Service de la Santé Publique du cantonal
☐ Autre : .... ☐ *Je ne sais pas/ Je ne souhaite pas répondre*

Support de distribution:

☐ Papier : ☐ brochure
☐ feuillet
☐ affiche
☐ autre : ....

☐ Electronique : ☐ brochure
☐ feuillet
☐ application
☐ autre : ....

☐ Autre : .... ☐ *Je ne sais pas/Je ne souhaite pas répondre*

Mode de distribution :

☐ Salles d'attente du médecin/de l'hôpital
☐ A la pharmacie
☐ Information transmise à domicile : ☐ par poste
☐ par E-mail
☐ par téléphone
☐ Médias : ☐ articles de journaux ou de magazines
☐ radio
☐ télévision
☐ Par internet : ☐ sites web
☐ réseaux sociaux
☐ Autre : ....
☐ *Je ne sais pas/ Je ne souhaite pas répondre*

4. Diffusion de la brochure sur le dépistage du cancer de la prostate

4a. Que pensez-vous du fait de diffuser la brochure sur le dépistage du cancer de la prostate aux citoyens entre 50 ans et 70 ans?

☐ Toujours souhaitable ☐ Parfois souhaitable
☐ Je ne peux me prononcer ☐ Rarement souhaitable
☐ Jamais souhaitable ☐ *Je ne sais pas/Je ne souhaite pas répondre*

Si votre réponse est positive, quelle.s serai.en.t selon vous la/les meilleure.s approche.s pour faire connaître la brochure sur le dépistage du cancer de la prostate aux citoyennes et citoyens?

Interlocuteur/distributeur :

☐ Distribution par le médecin

☐ Distribution par le pharmacien

☐ Distribution par d'autres professionnels (assistante médicale, infirmier, etc.)

☐ Distribution par le Service de la Santé Publique cantonal

☐ Autre :………………………………………………….

☐ *Je ne sais pas/Je ne souhaite pas répondre*

Support de distribution de la brochure:

☐ Version papier

☐ Version électronique

☐ Autre : ……………………………

☐ *Je ne sais pas/ Je ne souhaite pas répondre*

Mode de distribution de la brochure:

☐ Mise à disposition dans les salles d'attente du médecin/de l'hôpital

☐ Mise à disposition à la pharmacie

☐ Brochure envoyée à domicile : ☐ par poste ☐ par E-mail

☐ Mise à disposition sur internet : ☐ sites web ☐ réseaux sociaux

☐ Autre : ……………………………

☐ *Je ne sais pas/ Je ne souhaite pas répondre*

Commentaires :

……

……

……

……

……

……

Merci de donner votre avis sur la démarche au dos de ce livret

Conclusion – votre avis général

1. **Que pensez-vous du nombre de questions soumises en une fois ?**
   ☐ Beaucoup trop nombreuses ☐ Trop nombreuses
   ☐ Juste bien ☐ Pas assez nombreuses ☐ Pas d'avis

2. **Que pensez-vous du temps nécessaire pour répondre aux questionnaires?**
   ☐ Beaucoup trop long ☐ Trop long
   ☐ Juste bien ☐ Pas assez long ☐ Pas d'avis

3. **Quelle est votre impression générale de la démarche d'évaluation de la brochure par questionnaires à remplir à domicile?**
   ☐ Excellente ☐ Bonne
   ☐ Mauvaise ☐ Très mauvaise ☐ Pas d'avis

4. **Quelle est le meilleur support pour des questionnaires à remplir à domicile?**
   ☐ Papier ☐ Fichier électronique
   ☐ Questionnaire online ☐ Questions posées par téléphone
   ☐ Autre : .... ☐ Pas d'avis

5. **Comment avez-vous trouvé le format et la présentation des questionnaires (livret, taille des caractères d'écriture, etc.) ?**
   ☐ Excellent ☐ Très bien
   ☐ Bien ☐ Moyen
   ☐ Mauvais ☐ Très mauvais ☐ Pas d'avis

Points positifs/négatifs :

....

....

Suggestions :

....

....

....

Date : ....

Merci de votre participation active à nos efforts pour améliorer la transmission d'information sur le dépistage du cancer de la prostate aux citoyens concernés !

Version mai 2019

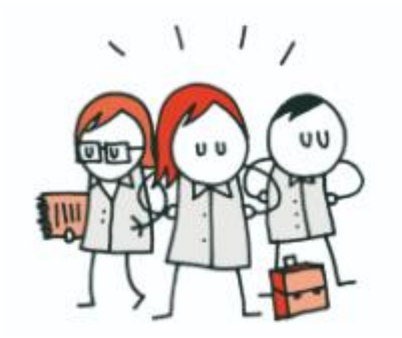

Votre avis compte…

Le Centre universitaire de médecine générale et santé publique (Unisanté) implique les citoyennes et citoyens afin d'améliorer la qualité de la documentation sur le dépistage des cancers, en collaboration avec la Fédération romande des consommateurs (FRC), Pro Senectute Vaud, la Faculté de biologie et de médecine (FBM) de l'UNIL, avec le soutien financier du Fonds de développement de la prévention et de la promotion de la santé du Service de la santé publique du canton de Vaud.

Nous souhaitons que la brochure « Le dépistage du cancer du poumon chez les fumeuses et fumeurs. Brochure d'information » soit compréhensible et soutienne les citoyennes et citoyens concerné·es dans leur prise de décision par rapport au dépistage du cancer du poumon.

Dans ce but, nous vous invitons à remplir les questionnaires du présent document :

- Le questionnaire 1 vise à évaluer la qualité de la brochure et son utilité dans la prise de décision informée des citoyennes et citoyens.
- La dernière page « Conclusion – votre avis général », au dos de ce document, vous permet de donner votre avis sur le projet.

En remplissant et nous retournant ces questionnaires, vous acceptez que les données soient utilisées de manière anonyme à des fins de recherche, de développement de brochures et de leur distribution aux citoyennes et citoyens. La participation est bénévole.

Nous vous serions reconnaissants de retourner ce document à l'aide de l'enveloppe jointe à notre courrier à :

Regula Cardinaux – Unisanté - Rue du Bugnon 44 - 1011 Lausanne
regula.cardinaux@unisante.ch – 021/314.20.14

Evaluation de la brochure :
« Le dépistage du cancer du poumon chez les fumeuses et fumeurs. Brochure d'information »

Afin de nous aider à améliorer la brochure « Le dépistage du cancer du poumon chez les fumeuses et fumeurs. Brochure d'information », **nous aimerions que vous répondiez attentivement aux questions ci-dessous en cochant les cases appropriées (une réponse par question) et en remplissant par un texte lorsque cela vous est proposé.**
Vous pouvez bien sûr vous aider de la brochure qui ne doit pas être diffusée, car en cours d'élaboration.

Si vous n'êtes plus fumeuse ou fumeur ou si vous ne l'avez pas été par le passé, nous vous demandons de répondre aux questions comme si vous deviez aviser ou prendre la décision avec une ou un de vos proches.

Certains termes employés pour désigner des personnes sont pris au sens générique ; ils ont à la fois valeur d'un féminin et d'un masculin.

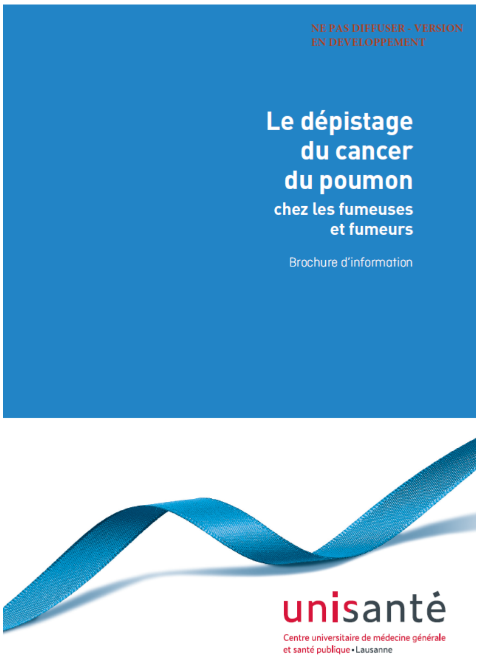

CONNAISSANCES

1. **Quelle proportion des cancers du poumon est causée par le tabac ?**
☐ 50%
☐ 75%
☐ 90%
☐ 99%
☐ *Je ne sais pas/ Je ne souhaite pas répondre*

2. **A qui le médecin recommande-t-il un test de dépistage du cancer du poumon ?**
☐ A toute personne en bonne santé
☐ A une personne avec une exposition importante au tabac qui est en bonne santé
☐ A une personne en consultation avec des symptômes suspect d'un cancer du poumon
☐ *Je ne sais pas/ Je ne souhaite pas répondre*

3. **Pour une personne avec une exposition importante au tabac, à partir de quel âge les médecins parlent-ils du test de dépistage du cancer de la prostate ?**
☐ 35 ans
☐ 45 ans
☐ 55 ans
☐ 65 ans
☐ *Je ne sais pas/ Je ne souhaite pas répondre*

4. **Si un résultat de scanner des poumons est anormal, cela signifie-t-il toujours que la personne a un cancer du poumon ?**
☐ Oui
☐ Non
☐ *Je ne sais pas/ Je ne souhaite pas répondre*

5. **Quelle est l'importance d'un résultat 'indéterminé' ?**
☐ Il faut systématiquement faire une biopsie d'un nodule au poumon
☐ La personne devra répéter un scanner des poumons dans 3-4 mois
☐ La personne fait son prochain scanner dans 1 à 2.5 ans, comme d'habitude
☐ *Je ne sais pas/ Je ne souhaite pas répondre*

6. **La dose de radiation reçue lors d'un scanner de dépistage dite 'low-dose' (faible dose) est :**
   ☐ Moins importante qu'un vol aérien
   ☐ Moins importante qu'une mammographie
   ☐ Moins importante que l'irradiation moyenne naturelle sur une année
   ☐ *Je ne sais pas/ Je ne souhaite pas répondre*

7. **Une découverte fortuite est la découverte d'anomalies autre qu'un cancer du poumon, passées inaperçues jusqu'à ce jour, lors d'un scanner de dépistage. Les découvertes fortuites :**
   ☐ Nécessitent souvent des examens supplémentaires
   ☐ Amènent presque toujours un bénéfice au patient
   ☐ Sont le plus souvent des cancers dans d'autres organes (ex : thyroïde)
   ☐ *Je ne sais pas/ Je ne souhaite pas répondre*

8. **Sur 250 personnes qui <u>ne</u> font <u>pas</u> le test de dépistage et dont on observe les conséquences durant 7 ans, environ combien mourront d'un cancer des poumons ?**
   ☐ Aucune
   ☐ 1 personne
   ☐ 3 personnes
   ☐ 4 personnes
   ☐ 14 personnes
   ☐ 16 personnes
   ☐ *Je ne sais pas/ Je ne souhaite pas répondre*

9. **Sur 250 personnes qui font le test de dépistage du cancer du poumon par scanner et dont on observe les conséquences durant 7 ans, environ combien mourront d'un cancer des poumons ?**
   ☐ Aucune
   ☐ 1 personne
   ☐ 3 personnes
   ☐ 4 personnes
   ☐ 14 personnes
   ☐ 16 personnes
   ☐ *Je ne sais pas/ Je ne souhaite pas répondre*

ATTITUDE ET INTENTION

10. Sur une échelle de 1 à 5 (1 étant le plus négatif et 5 le plus positif), que pensez-vous de chacune de ces six déclarations sur le dépistage du cancer du poumon?

Pour moi, faire le dépistage du cancer du poumon …

	1	2	3	4	5	
**10a.** …est une mauvaise chose	◯	◯	◯	◯	◯	…n'est pas une mauvaise chose
**10b.** …n'est pas bénéfique	◯	◯	◯	◯	◯	…est bénéfique
**10c.** …a des inconvénients	◯	◯	◯	◯	◯	…n'a pas d'inconvénients
**10d.** …n'est pas une bonne chose	◯	◯	◯	◯	◯	…est une bonne chose
**10e.** …n'a pas d'avantages	◯	◯	◯	◯	◯	…a des avantages
**10f.** …n'est pas important	◯	◯	◯	◯	◯	…est important

11. Mes intentions par rapport au dépistage du cancer du poumon :

Si vous n'êtes pas fumeur·se, quelle recommandation auriez-vous pour un proche ?

- ☐ J'ai la ferme intention d'effectuer un dépistage du cancer du poumon
- ☐ Je vais probablement effectuer un dépistage du cancer du poumon
- ☐ Je ne suis pas sûr d'effectuer un dépistage du cancer du poumon
- ☐ Je ne vais probablement pas effectuer un dépistage du cancer du poumon
- ☐ J'ai la ferme intention de ne pas effectuer un dépistage du cancer du poumon
- ☐ *Je ne sais pas/Je ne souhaite pas répondre*

		Oui	Non
**S**ûr	Etes-vous certain de ce qui constitue le meilleur choix pour vous ?	☐	☐
**U**tilité de l'information	Est-ce que vous connaissez les bénéfices et risques de chacune des options	☐	☐
**R**isques-bénéfices à balancer	Avez-vous le sentiment de savoir ce qui est le plus important pour vous à l'égard des risques et bénéfices ?	☐	☐
**E**ncouragement	Avez-vous suffisamment de soutien pour faire votre choix ?	☐	☐

The SURE Test © Connor and Légaré, 2008

INQUIETUDE ET REGRET

12. A quel point êtes-vous inquiet à l'idée de pouvoir avoir un cancer du poumon ?
Si vous n'êtes pas fumeur·se, quelle inquiétude auriez-vous pour un proche ?

Pas du tout inquiet	*Un peu inquiet*	*Assez inquiet*	*Très inquiet*	*Je ne sais pas/Je ne souhaite pas répondre*
☐	☐	☐	☐	☐

13. A quel degré approuvez-vous l'affirmation suivante :
Si vous n'êtes pas fumeur·se, que penseriez-vous pour un proche ?

a. **« Il est possible que plus tard je regrette d'avoir fait un dépistage » ?**
☐ J'approuve fortement ☐ J'approuve ☐ Je ne peux me prononcer
☐ Je n'approuve pas ☐ Je n'approuve pas du tout
☐ *Je ne sais pas/Je ne souhaite pas répondre*

b. **« Il est possible que plus tard je regrette de ne pas avoir fait un dépistage » ?**
☐ J'approuve fortement ☐ J'approuve ☐ Je ne peux me prononcer
☐ Je n'approuve pas ☐ Je n'approuve pas du tout
☐ *Je ne sais pas/Je ne souhaite pas répondre*

EVALUATION DE LA BROCHURE D'INFORMATION

14. Avez-vous lu la brochure « Le dépistage du cancer du poumon » ?
☐ En partie ☐ Au complet ☐ *Je ne souhaite pas répondre*

15. Avez-vous discuté du contenu de la brochure avec un professionnel de la santé ?
☐ Non ☐ Oui, avec un médecin ☐ Oui, avec un·e pharmacien·ne
☐ Oui, avec un autre professionnel de la santé ☐ *Je ne souhaite pas répondre*

16. Environ combien de temps avez-vous pris pour lire la brochure « Le dépistage du cancer du poumon » ?
................minutes

17. A quel point les informations contenues dans la brochure étaient-elles nouvelles pour vous ?
☐ Aucune information nouvelle ☐ Peu d'informations nouvelles
☐ Beaucoup d'informations nouvelles ☐ Entièrement nouvelles
☐ *Je ne sais pas/Je ne souhaite pas répondre*

18. Comment caractérisez-vous la taille de la brochure qui était nouvelle pour vous ?

☐ Vraiment trop longue ☐ Un peu trop longue ☐ Juste bien
☐ Un peu trop courte ☐ Vraiment trop courte
☐ *Je ne sais pas/Je ne souhaite pas répondre*

a. A quel point avez-vous trouvé la brochure neutre par rapport au dépistage ?

☐ Clairement en faveur du dépistage ☐ Un peu en faveur du dépistage
☐ Complètement neutre ☐ Un peu en défaveur du dépistage
☐ Clairement en défaveur du dépistage
☐ *Je ne sais pas/Je ne souhaite pas répondre*

19. A quel degré approuvez-vous l'affirmation suivante :
« J'ai trouvé l'information de la brochure claire et simple à comprendre » ?

☐ J'approuve fortement ☐ J'approuve
☐ Je ne peux me prononcer ☐ Je n'approuve pas
☐ Je n'approuve pas du tout ☐ *Je ne sais pas/Je ne souhaite pas répondre*

20. A quel degré approuvez-vous l'affirmation suivante :
« J'ai trouvé la brochure utile pour prendre ma décision par rapport au dépistage du cancer du poumon »

Si vous n'êtes pas fumeur·se, que penseriez-vous pour un proche ?

☐ J'approuve fortement ☐ J'approuve
☐ Je ne peux me prononcer ☐ Je n'approuve pas
☐ Je n'approuve pas du tout ☐ *Je ne sais pas/Je ne souhaite pas répondre*

21. A quel degré approuvez-vous l'affirmation suivante :
« Je recommanderais cette brochure à des personnes qui réfléchissent au dépistage du cancer du poumon » ?

☐ J'approuve fortement ☐ J'approuve
☐ Je ne peux me prononcer ☐ Je n'approuve pas
☐ Je n'approuve pas du tout ☐ *Je ne sais pas/Je ne souhaite pas répondre*

22. A quel degré approuvez-vous l'affirmation suivante :
« Je fais confiance aux informations présentées dans la brochure » ?

☐ J'approuve fortement ☐ J'approuve
☐ Je ne peux me prononcer ☐ Je n'approuve pas
☐ Je n'approuve pas du tout ☐ *Je ne sais pas/Je ne souhaite pas répondre*

23. Ajouter une page à la brochure sur les facteurs de risques et la prévention des cancers en général serait :

☐ Très utile ☐ Utile ☐ Je ne peux me prononcer ☐ Pas utile
☐ Pas du tout utile ☐ *Je ne sais pas/ Je ne souhaite pas répondre*

DONNEES PERSONNELLES

24. Quel est votre année de naissance ?

19 .... ☐ *Je ne souhaite pas répondre*

25. Etes-vous ?

☐ Un homme ☐ Une femme ☐ Autre ☐ *Je ne souhaite pas répondre*

26. Quel est votre pays d'origine ? ....

27. Est-ce que vous êtes ou étiez fumeur·se ?

☐ Oui ☐ Non ☐ *Je ne souhaite pas répondre*

28. Fumez-vous, même occasionnellement ?

☐ Oui ☐ Non ☐ *Je ne souhaite pas répondre*

29. A quel âge avez-vous commencé à fumer régulièrement ?

A .... ans

30. Si vous fumez, que fumez-vous ? (plusieurs réponses possibles)

☐ Des cigarettes
☐ Des cigares
☐ Des cigarillos
☐ La pipe
☐ La pipe à eau, chicha, narguilé
☐ Des cigarettes électroniques avec nicotine
☐ Des cigarettes électroniques sans nicotine
☐ Des produits de tabac chauffés comme iQOS (Pax, etc.)

31. Si vous ne fumez pas maintenant, avez-vous déjà fumé pendant plus de 6 mois régulièrement ?

☐ Oui ☐ Non ☐ *Je ne sais pas/ Je ne souhaite pas répondre*

32. Si vous avez arrêté de fumer, quand était-ce ?

☐ Dans les 12 derniers mois
☐ Il y a 1 an jusqu'à moins de 5 ans
☐ Il y a 5 ans jusqu'à moins de 15 ans
☐ Il y a 15 ans ou plus
☐ *Je ne sais pas/ Je ne souhaite pas répondre*

33. Si vous fumez ou avez fumé, avez-vous déjà fait un dépistage du cancer du poumon ?

☐ Oui ☐ Non ☐ *Je ne sais pas/ Je ne souhaite pas répondre*

a. Si oui, quand ?

Mois .... Année ....

b. Était-ce au cours des trois derniers mois ?

☐ Oui ☐ Non ☐ *Je ne sais pas/ Je ne souhaite pas répondre*

c. Si oui, pour quelle raison avez-vous effectué ce dépistage la dernière fois. Était-ce :

☐ A titre préventif ou de « check up », sans troubles ou symptômes auparavant
☐ A titre de contrôle suite à des douleurs ou à des symptômes
☐ A titre de contrôle suite à un test précédent qui avait révélé quelque chose
☐ Autre raison : ....
☐ *Je ne sais pas/Je ne souhaite pas répondre*

34. Avez-vous un médecin de famille personnel ? C'est-à-dire un médecin chez lequel vous pouvez vous rendre pour la plupart de vos problèmes de santé ?

☐ Oui ☐ Non ☐ *Je ne souhaite pas répondre*

a. Si oui, au cours des douze derniers mois, combien de fois vous êtes-vous rendu chez votre médecin de famille (médecin généraliste) ? Si vous ne savez pas exactement, estimez

Nombre de fois : ....

☐ *Je ne sais pas/ Je ne souhaite pas répondre*

35. Comment est votre état de santé ?

☐ Très bon ☐ Bon ☐ Assez bon ☐ Mauvais ☐ Très mauvais
☐ *Je ne sais pas/ Je ne souhaite pas répondre*

36. Quel est le plus haut degré de formation que vous avez achevée ?

☐ Scolarité obligatoire ☐ Ecole de métier / apprentissage
☐ Maturité/baccalauréat ☐ Ecole professionnelle supérieure
☐ Haute école spécialisée/ pédagogique ☐Université
☐ Autre : ....
☐ Aucune ☐ *Je ne souhaite pas répondre*

37. Quel est le métier exercé ? ....

38. J'ai eu connaissance du projet par :

☐ Fédération romande des consommateurs ☐ Pro Senectute Vaud
☐ Université de Lausanne – patients simulés
☐ Unisanté (anciennement PMU)
☐ Autre : ....

Conclusion – votre avis général

1. **Que pensez-vous du nombre de fois que nous vous avons sollicité pour ce projet ?**

☐ Beaucoup trop nombreux ☐ Trop nombreux
☐ Juste bien ☐ Pas assez nombreux ☐ Pas d'avis

2. **Que pensez-vous de la durée du projet ?**

☐ Beaucoup trop long ☐ Trop long ☐ Juste bien
☐ Pas assez long ☐ Pas d'avis

3. **Quelle est votre impression générale du projet ?**

☐ Excellente ☐ Bonne ☐ Mauvaise
☐ Très mauvaise ☐ Pas d'avis

4. **Quels sont les axes d'amélioration possibles pour ce type de projet participatif ?**

………
………
………
………
………
………
………
………
………
………
………
………
………
………
………

Date : ……………………………………………

Merci de votre participation active à nos efforts pour améliorer la transmission d'information sur le dépistage du cancer du poumon aux citoyennes et citoyens !

Version janvier 2020
